# Dynamic nanoindentation testing: is there an influence on a material’s hardness?

**DOI:** 10.1080/21663831.2017.1331384

**Published:** 2017-11-03

**Authors:** A. Leitner, V. Maier-Kiener, D. Kiener

**Affiliations:** ^a^ Department Materials Physics, Montanuniversität Leoben, Leoben, Austria; ^b^ Department Physical Metallurgy and Materials Testing, Montanuniversität Leoben, Leoben, Austria

**Keywords:** Nanoindentation, dynamic indentation testing, mechanical properties, strain-rate sensitivity

## Abstract

Modern nanoindentation devices are capable of dynamic experimentations, which allow us to exploit instrumented hardness tests extensively. Beside the assets of recording mechanical properties continuously over displacement, there are ongoing debates whether the superimposed force alters the material’s hardness. We will show for a broad range of materials that significant hardness differences are noted between dynamic and static tests, even for large displacements. Those mainly result from a changing indentation strain-rate during the hold segment at peak load. This fact must be implicitly considered in studies using static indentation tests to guarantee comparability of obtained nanoindentation hardness values and derived quantities.

## GRAPHICAL ABSTRACT

**Figure:**
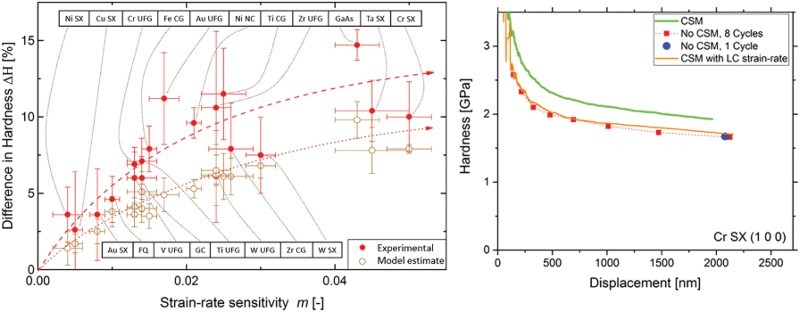

**IMPACT STATEMENT**

A mismatch between static and dynamic nanoindentation hardness has been identified to result from strain-rate deviations during hold segments in static nanoindentation tests, in particular affecting rate-sensitive materials.

## Introduction

Hardness as a mechanical property is a convenient parameter to assess a material’s strength, since it is intuitive that a harder material will be able to penetrate a softer material, but not vice versa [1]. Nowadays, it is possible to maximize the output of instrumented indentation experiments, since the contact stiffness and thereby also the material’s hardness and Young’s modulus can be obtained continuously over indentation depth [2–6]. This depth-dependent data can further be useful to state the reliability of measurements or to correct for thermal drift during long-time test [7,8], since the used dynamic technique is less sensitive to thermal drift problems [5,9]. To continuously access the stiffness of the material, it is unavoidable to manipulate the force signal by superimposing an oscillating force with diminutive amplitude where the phase shift between load and displacement signal will allow us to calculate the elastic stiffness [2,3,10], often referred to as continuous stiffness measurement (CSM) or dynamic indentation testing. While the technical implementation of this oscillation is current state-of-the-art, there is still an ongoing debate on whether this superimposed signal influences the mechanical properties of materials. Therefore, several comprehensive studies investigated the impact of amplitude level and frequency on the obtained mechanical properties [10–16]. Differences in hardness values for low indentation depths with comparable large oscillation amplitudes have been noted several times [13,15,16]. They occur when the superimposed force amplitude is significant compared to the indentation depth and when measurements are executed with rather low oscillation frequencies, indicating partial loss of contact. However, discrepancies are also conceivable at high loads and displacements and should be examined to exclude contact issues. Still, comparative and systematic studies performed on identical samples and nanoindentation devices are lacking in literature. Within the present study, we investigate the differences among nanoindentation testing methods with and without CSM with focus on deep indentations, thus, excluding possible surface effects. We demonstrate for a broad selection of materials that the hardness difference is mainly dependent on the material inherent strain-rate sensitivity *m* of the tested sample rather than the used indentation technique. In future, this matter needs to be consistently considered in the data analysis of static nanoindentation tests.

## Experimental

To assess the influence of lattice type and microstructure, materials listed in Table 1 were selected for the investigations. Additionally, this selection covers large intervals of hardness *H*, Young’s modulus *E* and strain-rate sensitivity *m* values. Ultra-fine-grained materials were fabricated by High Pressure Torsion [17–19] and nanocrystalline (NC) Ni by electrodeposition [20], while melt grown single crystals and coarse grained materials were tested as received from suppliers. The microstructures were investigated and analyzed by optical microscope imaging, electron backscatter diffraction or X-Ray diffraction. Additionally, fused quartz (FQ) and glassy carbon (GC) were tested to examine whether the impact of CSM can also be noted on amorphous ceramic samples. Details of used specimens can be obtained from former studies [8,15,21,22].

**Table 1.** Investigated material selection, covering face-centered cubic (FCC), body-centered cubic (BCC) and hexagonal closed packed (HCP) metals as well as FQ, GC and GaAs.

Material	Lattice type	Microstructure	Young’s modulus, *E* (GPa)	Strain-rate sensitivity (*m*)
Au	FCC	SX (1 0 0)	75 ± 1	0.008 ± 0.001
Au	FCC	UFG 250 nm	79 ± 2	0.017 ± 0.002
Cu	FCC	SX (1 1 1)	121 ± 2	0.005 ± 0.001
Ni	FCC	SX (1 0 0)	196 ± 2	0.003 ± 0.002
Ni	FCC	NC 30 nm	205 ± 2	0.021 ± 0.001
V	BCC	UFG 200 nm	144 ± 1	0.013 ± 0.001
Ta	BCC	SX (1 0 0)	169 ± 1	0.045 ± 0.005
Fe	BCC	CG 100 µm	195 ± 2	0.015 ± 0.001
Cr	BCC	SX (1 0 0)	293 ± 5	0.050 ± 0.003
Cr	BCC	UFG 300 nm	291 ± 2	0.013 ± 0.001
W	BCC	SX (1 0 0)	397 ± 7	0.030 ± 0.002
W	BCC	UFG 890 nm	407 ± 9	0.024 ± 0.002
Zr	HCP	CG 45 µm	109 ± 1	0.026 ± 0.003
Zr	HCP	UFG	127 ± 2	0.024 ± 0.002
Ti	HCP	CG 55 µm	125 ± 1	0.025 ± 0.003
Ti	HCP	UFG	192 ± 3	0.014 ± 0.002
GaAs	Zinc blende	SX	114 ± 1	0.043 ± 0.003
Glassy C	–	amorphous	34 ± 1	0.014 ± 0.002
FQ	–	amorphous	72 ± 1	0.010 ± 0.001

Note: The Young’s moduli were obtained from CSM measurements, and *m* values from nanoindentation jump tests. UFG Zr contains omega phase.

Indentation tests were conducted on a Keysight G200 Nanoindenter equipped with CSM option and a diamond Berkovich indenter tip to obtain *H* and *E* by the conventional analysis firstly proposed by Oliver and Pharr [3]. For each material two different types of tests were executed. On the one hand, constant strain-rate CSM measurements with a frequency of 45 Hz and a displacement amplitude of 2 nm were performed to determine the mechanical properties continuously over displacement. The indentation strain-rate 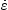 was set to 0.05 s^−1^. On the other hand, tests with multiple unloading sequences (8 unloading regimes distributed over 2500 nm of indentation depth) without CSM, denoted as load-controlled (LC) tests in this study, also allow us to extract eight pairs of *E* and *H* within one single indent. The hold time at maximum load was set to 5 s, and the time to load was set to 15 s, resulting in about same indentation strain-rate for all materials [23]. LC and CSM measurements were executed consecutively with the same tip area function to exclude any errors from different tip shapes and calibrations, which could potentially falsify the investigated effect. Additionally, the mechanical properties obtained by CSM were analyzed at the same displacement as the corresponding LC measurements to avoid influences of indentation size effects [24]. Moreover, single LC experiments without CSM were executed for Cr SX and Ni SX to check whether thermal drift or multiple load–unload segments are a potential source of error. At least five indents were performed for each set of parameters. For all materials a maximum displacement of 2500 nm was used, with the exception of FQ, UFG Cr, UFG W and GaAs, where the force limit of 660 mN was reached earlier (*h*_max,FQ_ = 2400 nm, *h*_max,UFG Cr_ = 2470 nm, *h*_max,UFG W_ = 2000 nm, *h*_max,GaAs_ = 2300 nm). Thermal drift did not exceed 0.1 nm/s for any considered indentation. Occurring hardness differences for different test methods were obtained at maximum common displacement. Strain-rate jump tests were conducted to determine *m*, following the work of Maier et al. [25]. An overview of the used samples and their properties is given in Table 1.

## Results and discussion

**Figure 1.**
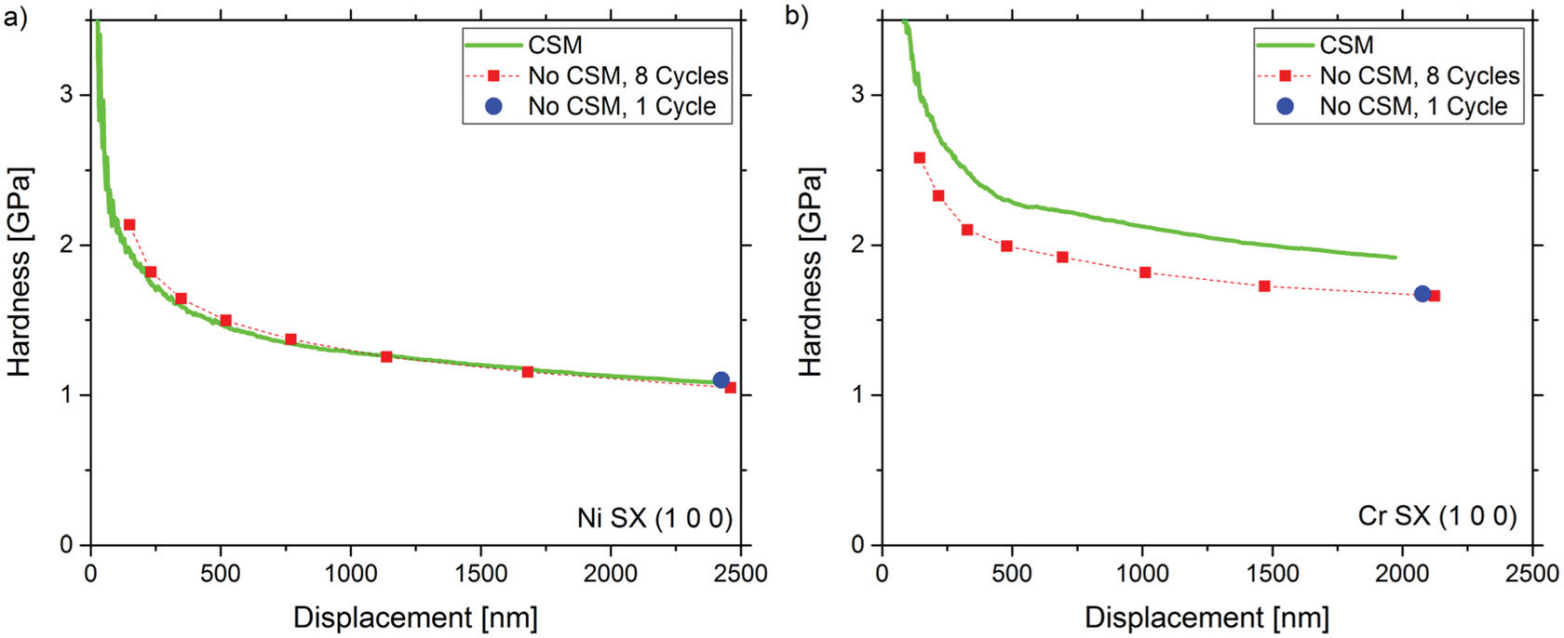
Comparison of hardness—displacement profiles for (a) Ni SX and (b) Cr SX for tests with and without CSM. Evidently, the different techniques result in significantly different *H* levels for Cr SX but not for Ni SX.

Two representative hardness profiles of the different measurement types for Ni SX and Cr SX are displayed in Figure 1. Both materials show a distinct indentation size effect as expected for SX [24]. It can evidently be noted that while for Ni SX the hardness values for all test types coincide over the entire displacement range, a significant deviation between LC and CSM data appears for Cr SX. Multiple loading tests do not differ from single loading tests at maximum displacement, indicating that drift can be excluded as a potential source of error, as the multiple holding segments increase the test time and would amplify any drift influences.

**Figure 2.**
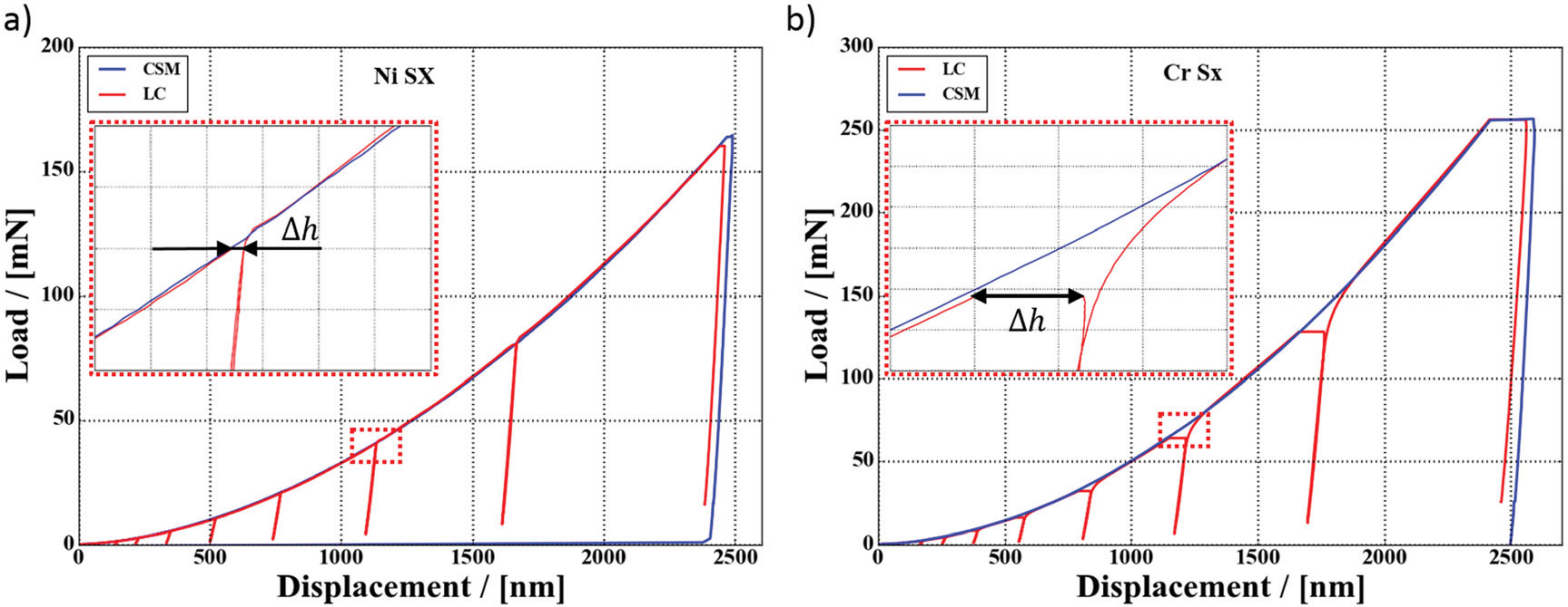
Load—displacement curves of LC (multiple unloading) and CSM measurements on Ni SX and Cr SX demonstrate that the hardness difference originates from the dwell segments of the LC tests (see the insets), while the plastic loading curves match up well.

One could think that the crystal structure (FCC versus BCC in Figure 1) may be the decisive factor, but also measurements on FCC metals with refined microstructures show a significant hardness difference between CSM and LC tests. To analyze this behavior in detail, we overlay the two load–displacement curves from CSM and LC measurements on top of each other, as shown in Figure 2. The plastic loading curves match well; thus the mismatch in hardness for Cr SX must originate from the holding regime. The increase in the displacement *Δh* during these hold segments is referred to as indentation creep, the amount of which in turn is linked to the strain-rate sensitivity of the materials. This suggests that the effect may result from a changing strain-rate during the hold segment, as the effect is less pronounced for rate-insensitive Ni SX in Figure 2(a). Before this circumstance is discussed in detail, the definition of the indentation strain-rate shall briefly be revisited.

**Figure 3.**
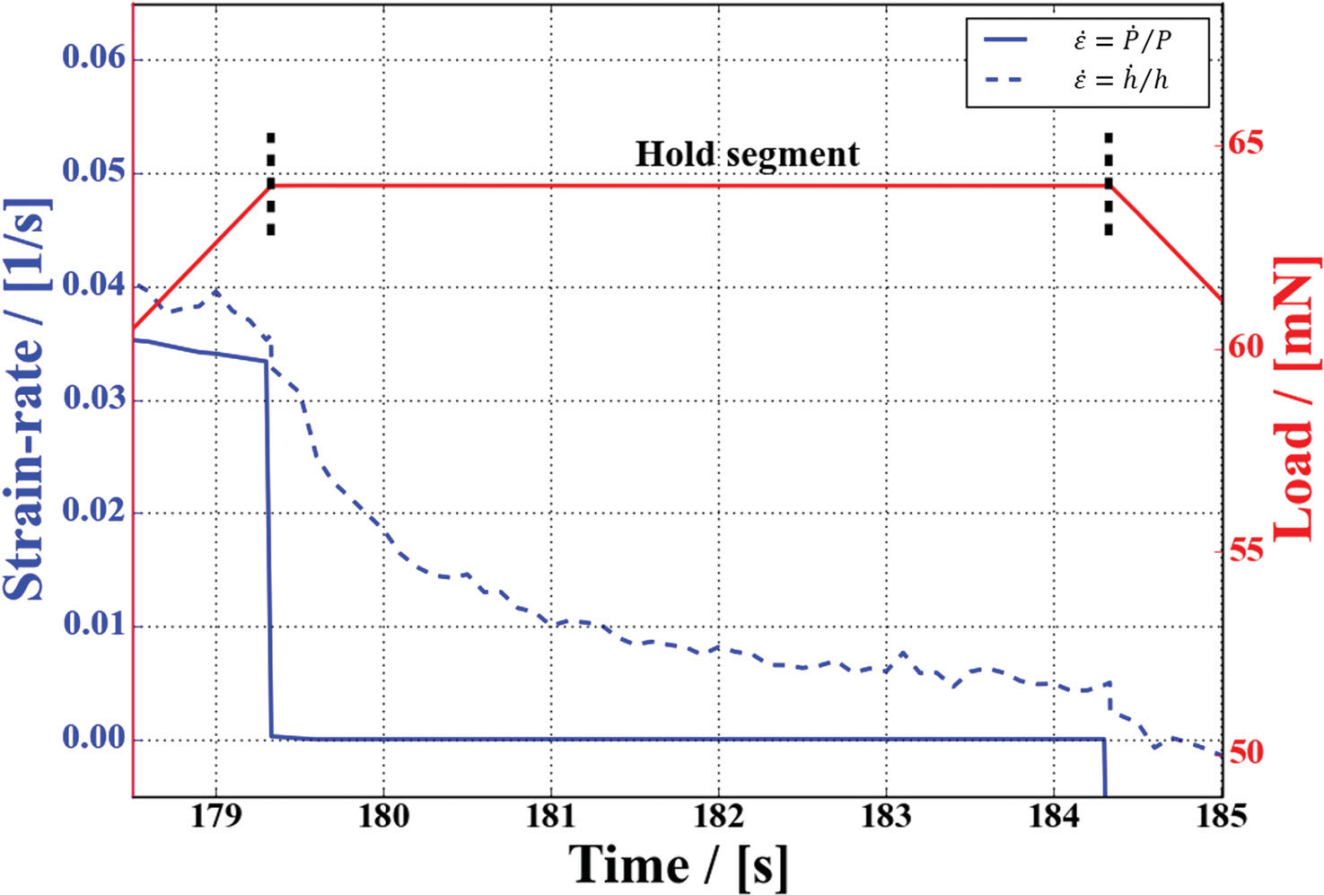
Development of the actual indentation strain-rate defined as 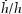, in contrast to the approximate solution 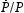 for a hold segment of a quasi-static indentation experiment. Evidently, the approximate 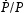 solution breaks down during constant load regimes.

Since the indentation strain is conventionally assumed to be only dependent on the tip geometry, self-similar pyramidal tips, such as the used Berkovich tip, introduce the same strain independent of the indentation depth. Hence, the strain-rate is not defined by the temporal increase of the strain itself but as the expansion of the self-similar plastic zone through the material. As this parameter is experimentally not accessible, alternatively the indentation strain-rate can be defined as [26](1)
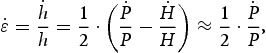
where 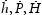 are the time derivatives of the displacement, load and hardness, respectively. The fact that this study investigates differences at high displacements is essential for the accuracy of this work since tip imperfections, often noted for low depths, lead to deviations from the self-similar geometry and could unpredictably alter the physical strain-rate [27]. However, the approximation in the last step is strictly spoken valid only for materials with constant hardness over displacement or materials where the hardening rate 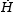 is negligibly low compared to the corresponding hardness. Figure 3 shows the difference of the evaluated strain-rate during the hold segment by either the definition of displacement or definition of load, respectively. Particularly during the dwell segment the approximation breaks down as the load is held constant and consequently the strain-rate converges to zero (see Figure 3). Nevertheless, the right part of Equation (1) is generally used since this parameter is easier to trigger for common nanoindentation devices. During the hold segment the strain-rate will be dominated by the change of hardness or the change of displacement, respectively, clearly making the definition 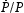 unfeasible in this regime.

**Figure 4.**
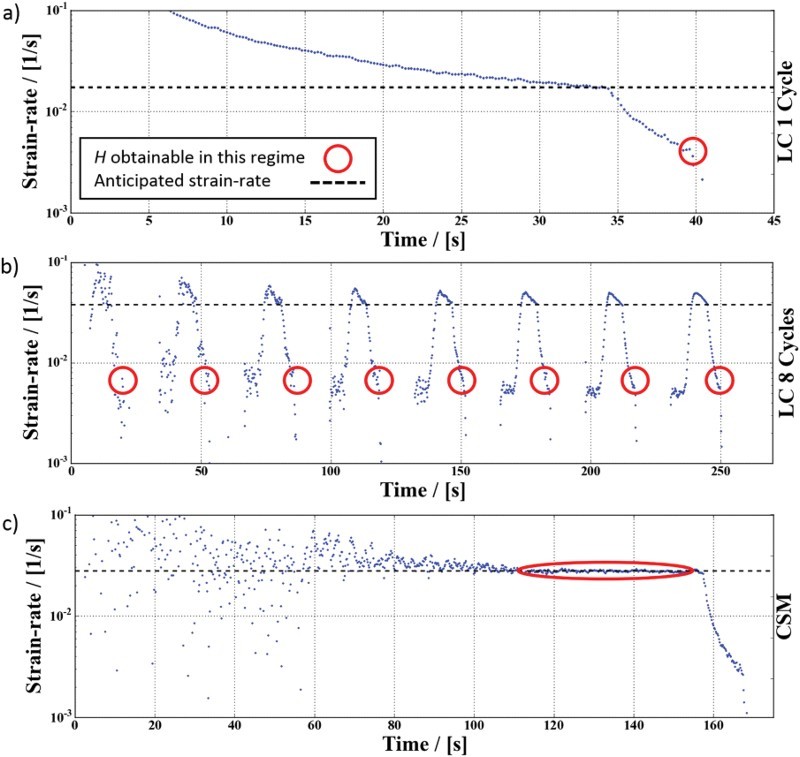
(a)–(c) Strain-rate profiles of three common nanoindentation techniques for Cr SX. The red circles indicate the range that can be used to obtain mechanical properties by the Oliver and Pharr analysis. Note that for dynamic (CSM) testing (c) the anticipated strain-rate is achieved, while for static (LC) tests (a, b) it differs by one order of magnitude.

As evident from Figure 3, the strain-rate at which the hardness value is evaluated has to be inevitably considered. Following the analysis of Oliver and Pharr [3], the material stiffness is required to compute the real area in contact to account for the elastic sink-in during indentation. For LC measurements, this requires the unloading curve and therefore the mechanical properties can only be determined at the point of unloading, which is assigned to the corresponding strain-rate at this point. As seen from Figures 3 and 4, this strain-rate can be significantly different from the strain-rate during loading and naturally varies in dependence of the hold time. This behavior enables to obtain *m* in nanoindentation creep tests [7,26,28,29]. In contrast, the CSM technique allows to determine the hardness at the set 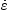 value during loading. Figure 4(a)–(c) give a detailed view of the strain-rate profiles for the conducted testing methods, showing that for the case of Cr SX the strain-rate difference turns out to be more than one order of magnitude for the 5 s hold segments.

If we assume that the hardness deviations between the testing protocols emerge from the strain-rate changes, we can calculate the expected hardness difference if the strain-rate sensitivity is known. Using the common power-law behavior for the strain-rate sensitivity dependent hardness [30–32], one derives(2)
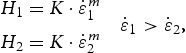

(3)
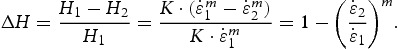



**Figure 5.**
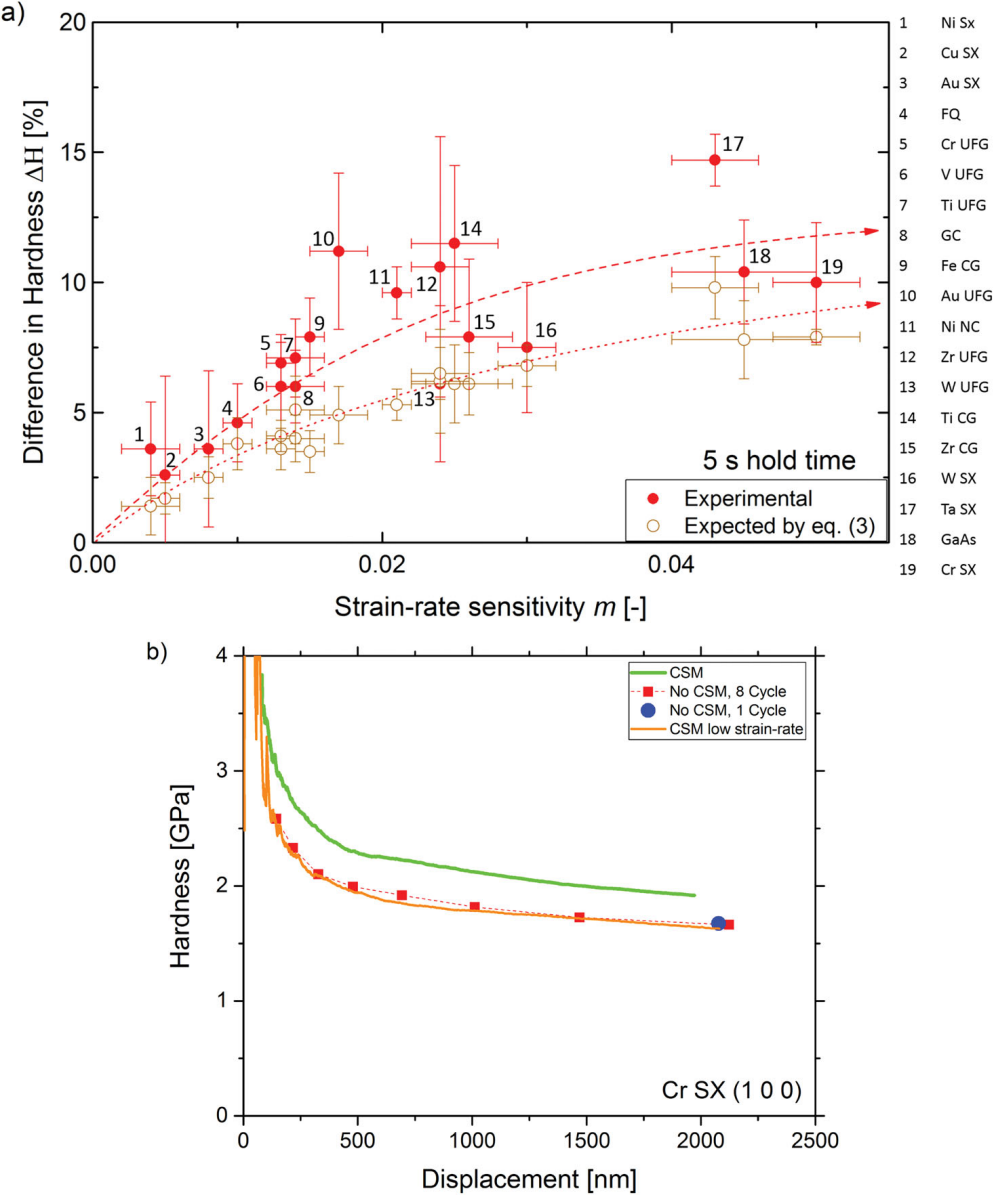
(a) Experimental and theoretical hardness difference as expected from Equation (3) between static and dynamic tests in dependence of the strain-rate sensitivity. (b) Low strain-rate CSM experiments confirm that the hardness difference is mainly rooted in differing strain-rates used to determine the hardness.

According to Equation (3), this should lead to an increasing hardness difference between LC and CSM tests with increasing strain-rate sensitivity. This is evident in Figure 5(a), where the difference between CSM and LC hardness is shown in dependence of the strain-rate sensitivity of the materials. The closed symbols depict the experimentally measured hardness difference, while the open symbols represent calculated results from Equation (3). Therefore actual strain-rates were determined at the depths used for the analysis with the according *m* values of Table 1. It is noted that the displayed data in Figure 5(a) are for a constant hold time of 5 s, meaning that the ratio 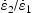 varies depending on the material. The data follow the predicted behavior from Equation (3), strain-rate-insensitive materials such as FCC SX show only slight differences, while highly rate-dependent materials such as BCC or fine-grained structures show large differences. Cr SX was used to experimentally prove whether this strain-rate-effect is decisive. The strain-rate during the hold regime of the LC test (Figure 3) was determined and resulted in a value of 0.005 s^−1^ at the point of unloading. Subsequently this strain-rate was set in a CSM test. Figure 5(b) shows fairly good agreement between the hardness—depth profiles for the LC experiments and the low strain-rate CSM experiments, which confirms the dominance of the described strain-rate-effect. Considering the actual strain-rates from the load–displacement data rather than the preset values, the difference in hardness reduces to an average value of 2.5%. Still, CSM measurements show slight but noticeably higher hardness values compared to LC measurements, which might result from a weak hardness dependence of *m*, as known from nanoindentation creep experiments [7,8,29]. This fairly small systematic difference could also emerge due to differences in the loaded material structure between LC, where a stable microstructure is unloaded, and CSM, where the microstructural evolution is based on a dynamic equilibrium structure.

## Summary and conclusion

To the best knowledge of the authors, hardness differences between static LC and dynamic CSM tests are a matter of debate, but have never been systematically examined. Within this study, a broad spectrum of materials including FCC, BCC, HCP metals, semiconductor and amorphous materials has been investigated at high displacements, thus excluding any possible surface artifacts. As demonstrated here, LC results can significantly deviate from CSM measurements for strain-rate-sensitive materials. We confirm that this effect is mainly attributed to the changing strain-rate during dwell segments of static LC tests rather than to any changes in the material’s microstructure. Considering this matter considerably decreases the mismatch down to an average level of only 2.5%. It is tremendously important to consider this rate-effect; otherwise the hardness differences could misleadingly be ascribed to the force oscillation.

This finding implies broad-ranging consequences for indentation testing of rate-sensitive materials. It also exemplifies the challenges and requirements when comparing literature values from static nanoindentation testing techniques. Also, the determination of *m* by static nanoindentation constant strain-rate experiments is disputable. The specified strain-rates with the definition 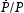 for LC tests do not represent the actual occurring strain-rate as long as peak hold times are part of the measurement protocol, which is the standard implementation. In fact, such data only indicate the upper limit of the strain-rate. Therefore, we strongly recommend obtaining the actual indentation strain-rate at the end of the hold segment. This can effortlessly and accurately be determined from the load–displacement data and guarantees accurate and comparable results.

However, still there remains a hardness gap with slightly higher *H* values for CSM measurements, which could conceivably be attributed to slightly differing microstructures between static and dynamic conditions or a slight hardness dependence *m*. In addition, the discussed effect has been investigated at rather large indentation depths, but does not necessarily depict the situation at shallow depths, where the displacement amplitude of the CSM signal is considerably large compared to the indentation depth [13,15,16]. In such a situation, the hardness value will be influenced additionally from changes in the contact stiffness, which will further complicate the identification of the underlying physical effects in a rate-sensitive material. Addressing this matter is, however, beyond the scope of the present work.
